# The role of IL-35 and IL-37 in breast cancer – potential therapeutic targets for precision medicine

**DOI:** 10.3389/fonc.2022.1051282

**Published:** 2022-11-22

**Authors:** Yuntao Ma, He Su, Xuyun Wang, Xiangdong Niu, Yang Che, Brett D. Hambly, Shisan Bao, Xiaopeng Wang

**Affiliations:** ^1^ Department of General Surgery, Gansu Provincial Hospital, Lanzhou, Gansu, China; ^2^ Centre for Healthy Futures, Torrens University Australia, Sydney, NSW, Australia

**Keywords:** breast cancer, IL-35, IL-37, therapeutic targets, anti-inflammatory

## Abstract

Breast cancer is still a major concern due to its relatively poor prognosis in women, although there are many approaches being developed for the management of breast cancer. Extensive studies demonstrate that the development of breast cancer is determined by pro versus anti tumorigenesis factors, which are closely related to host immunity. IL-35 and IL-37, anti-inflammatory cytokines, play an important role in the maintenance of immune homeostasis. The current review focuses on the correlation between clinical presentations and the expression of IL-35 and IL-37, as well as the potential underlying mechanism during the development of breast cancer *in vitro* and *in vivo*. IL-35 is inversely correlated the differentiation and prognosis in breast cancer patients; whereas IL-37 shows dual roles during the development of breast cancer, and may be breast cancer stage dependent. Such information might be useful for both basic scientists and medical practitioners in the management of breast cancer patients.

## Breast cancer

Breast cancer continues to attract considerable attention from basic scientists and clinicians around the world, mainly due to the high morbidity following diagnosis of this life-threatening cancer in women, in addition to being the leading cause of cancer death among women (1), despite extensive clinical and basic research over recent decades. Most breast cancers are in women, although <1% occur in males with an extremely poor prognosis (2). Following the expansion of annual breast cancer screening over recent years, > 90% of breast cancers are diagnosed in the US at an early stage without metastasis (3). The management of breast cancer varies, depending on the invasion and metastasis of the breast cancer. Surgical resection (lumpectomy) is usually applied to the patients without metastasis (4), followed by postoperative radiation. Systemic therapy for non-metastatic breast cancer is determined by subtype: patients with hormone receptor–positive tumours receive endocrine therapy, and a minority receive chemotherapy as well; patients with Erb-B2 receptor tyrosine kinase 2 (ERBB2)-positive tumours receive ERBB2-targeted antibody or small-molecule inhibitor therapy combined with chemotherapy; and patients with triple-negative tumours receive chemotherapy alone. The prognosis of breast cancers is closely related to the surface expression of key molecules, i.e. estrogen and progesterone receptors, and ERBB2 (HER2), defining the classification of breast cancers into three categories: ERBB2 negative (~70% of patients), ERBB2 positive (~15%-20%), and triple-negative (tumours lacking all 3 standard molecular markers; ~15%) (5).

Although there are very good postoperative precision medicines protocols for the management of breast cancer patients, based on preoperative treatment response (6), the prognosis of breast cancer patients with metastatic disease is still poor, particularly among triple-negative breast cancers, where the median survival is ~ 1 year *vs* approximately 5 years for the other 2 subtypes.

Two key contributing factors in the development of breast cancer are activation of oncogenes and de-activation of tumour suppressor genes (7). In addition, host immunity plays a critical role in the development of breast cancer, supported by the finding that there is a close correlation between tumour infiltrating lymphocytes (TILs) and a more-favourable prognosis in patients with early-stage triple-negative and HER2-positive breast cancer (8). TILs are often detected in breast cancer tissues, although their significance is subject to debate, i.e. are these lymphocytes beneficial or harmful during the development of breast cancer (9). TILs are mainly CD8^+^ cytotoxic T cells, CD4^+^ helper T cells and B cells in breast cancer (9). Activated CD8^+^ T cells have been shown to kill breast cancer cells directly *in vitro* (10) and in an animal model *in vivo* (11); whereas infiltrating CD4^+^ cells contribute to the host immunity against malignant cells indirectly *via* secreting cytokines, e.g. IL-6, TNF, IL-18 (12). However, it has been reported that some TILs, e.g. T regulatory (Treg) cells, which are CD4^+^/CD25^+^, are able to regulate host immunity *via* the IL-35 signalling pathway (13), which may contribute to immune escape of malignant cells.

In order to ensure self-protection during an inflammatory response, Treg cells are promoted by the PD-1 (programmed cell death-1 (PD-1)/programmed cell death-1 ligand 1 (PD-L1) pathway to inhibit self-reactive T cells, e.g. to prevent a deleterious autoimmune response. However, within the context of a malignancy, activation of the PD-1/PD-L1 pathway contributes to tumorigenesis and enhances progression of malignant cells. Subsequent clinical evidence has shown promising outcomes for the application of PD-1 and PD-L1 inhibition in triple negative breast cancer (14), supporting the involvement of host immunity during the development of this form of breast cancer.

More recently, the presence of a large number of infiltrating macrophages in cancer tissues, which are termed tumour associated macrophages (TAMs), has been investigated (15). TAMs have been shown to enhance cancer growth *via* upregulating PD-L1 expression (16). Importantly, the function of macrophages in oncogenesis is also debatable (17), i.e. some macrophages, classified as classically activated macrophage M1 cells, contribute to killing malignant cells by supporting T cell-mediated anti-tumour immune responses, by secreting pro-inflammatory cytokines, e.g. tumour necrosis factor (TNF) and nitric oxide; but other TAMs, alternatively activated M2 macrophages, promote proliferation of cancer cells and immunosuppression by the secretion of growth factors and anti-inflammatory cytokines (18). This discrepancy in TAM activity probably is due to different microenvironments that direct and determine the final terminal differentiation of TAMs from the original monocytes in the circulating blood (19). Importantly, a recent report has outlined a novel approach that can be used to re-educate TAMs to promote the specific targeting of malignant cells with promising outcomes, using bioengineering manipulation (20). Generally, there are abundant infiltrating leucocytes within a breast cancer, including lymphocytes, neutrophils and macrophages (21). More recent studies demonstrate that the role of TAMs is, however, controversial, particularly in relation to the terminal differentiation of macrophages from monocytes (22). Following macrophage differentiation, classically activated M1 macrophages express HLA-DR, CD80/86 and alternatively activated M2 macrophages express CD206, CD163, CD204 (18), based on their surface markers and functionalities. M1 macrophages are believed to typically exert anti-tumour functions, including directly mediated cytotoxicity, e.g. the release of TNF, IFN-γ, and free radicals (reactive oxygen species and nitric oxide), which contributes to direct killing of tumour cells. In contrast, M2 macrophages can promote proliferation and metastasis of tumour cells *via* enhancing the function of Treg cells and the subsequent inhibition of the T cell-mediated anti-tumour immune response, and the promotion of tumour angiogenesis, which leads to tumour progression (15). It has been demonstrated that IL-37 inhibits the maturation of M2 macrophages in hepatocellular carcinoma tissues (23) and inhibits angiogenesis (24).

## IL-35

IL-35, an anti-inflammatory cytokine that is a member of the IL-12 family, is important in maintaining host immune stability, particularly in chronic inflammatory conditions in humans, a concept that is supported by reports showing that dysregulated IL-35 is observed in autoimmune diseases, e.g. systemic lupus erythematosus (25), rheumatoid arthritis (26) and atherosclerosis (27). In relation to cancer, IL-35 promotes tumour growth *via* suppressing host anti-tumour immunity within the tumour microenvironment, due to its capacity to regulate the immune response (28). Activation of recepteur d’origine nantais (RON) receptor tyrosine kinase signalling in macrophages has been shown to be a key player in supporting a thriving mammary pro-tumour microenvironment through novel mechanisms including the augmentation of tumour cell properties through IL-35 by TAMs (29).

## IL-37

IL-37 belongs to the IL-1 superfamily (30). Constitutive expression of IL-37 is observed in leucocytes, epithelial cells and many other tissues including draining lymph nodes, normal or atherosclerotic arteries, lung and intestine (31, 32). IL-37 is classified as an anti-inflammatory cytokine, because it regulates the host immune response (33) *via* inhibition of innate (34) as well as adaptive immunity (35). IL-37 balances host immunity/inflammation in the microenvironment *via* competing with the activities of pro-inflammatory cytokines, under normal conditions (36–38). However, dysregulated/upregulated IL-37 expression is observed in autoimmune diseases, including rheumatoid arthritis (37), psoriasis, Grave’s disease, gestational diabetes mellitus (39), systemic lupus erythematosus and inflammatory bowel disease (38). Mechanistic investigation of IL-37 demonstrates that the role of IL-37 (33) is related to promoting differential polarisation of macrophages *via* promoting M2 towards M1 macrophage polarisation (23), as well as suppression of the maturation of dendritic cells in atherosclerotic plaque (40), thus effectively reducing the development of atherosclerosis (41), which has been confirmed in an IL-37 transgenic animal model (42). IL-37 has been shown to be protective during the development of a number of cancers, including hepatocellular carcinoma (43), colorectal cancer (44), non-small cell lung cancer, renal cell carcinoma and oral and cervical squamous cell carcinoma (45). Possible anti-tumour mechanisms of IL-37 include inhibition of both angiogenesis and tumour-promoting inflammation, and promotion of anti-tumour immunity (24). Paradoxically, high circulating levels of IL-37 have been shown to associated with decreased survival in patients with metastatic epithelial ovarian cancer (45), possibly due to a unique tumour microenvironment in this form of cancer. However, the authors did not measure IL-37 levels within the tumour itself, making comparison to other cancer studies difficult.

The role of IL-35 and IL-37 have been well reviewed in autoimmune diseases, e.g. rheumatoid arthritis, Hashimoto’s thyroiditis, allergic respiratory diseases and cardiovascular diseases (46). However, the involvement of IL-35 and IL-37 in breast cancer has not been rigorously reviewed.

## IL-35 in breast cancer

There is almost no constitutive IL-35 expression in normal breast tissue, but the expression of IL-35 is increased and inversely correlated with the differentiation of breast cancer, when detected using immunohistochemical labelled with both subunits of IL-35, i.e. anti- Epstein-Barr virus induced gene 3, and p35 antibodies (47). The source of IL-35 has been identified as breast cancer cells and TILs. Importantly, breast cancer-derived IL-35 is able to convert conventional T cells into suppressive induced Treg IL-35 producing cells (47). Thus, the data suggest that IL-35 acts in both a paracrine and autocrine fashion during the development of breast cancer by promoting breast cancer proliferation and invasion (47), and by suppressing host immunity against cancer cells at the cellular and humoral levels (48), which may involve suppressing direct T cell mediated and/or antibody-dependent cytotoxicity against cancer cells (49).

Consistently, an inverse correlation has been observed between IL-35 expression and overall survival of breast cancer patients (47), which further supports the role of IL-35 in enhancing breast cancer development directly and/or indirectly *via* promoting polarisation of TAMs, which eventually predominantly become M2 macrophages, an observation that will be further discussed in the following sections. The inverse correlation between IL-35 expression and survival/tumour differentiation of breast cancer patients demonstrates that IL-35 may be used as a predictive biomarker for post-operative breast cancer patients. It has further been demonstrated that breast cancer cells can produce IL-35 *in vitro* and can also be manipulated by the CRISPR/Cas9 system at the molecular and cellular levels (47). Such findings suggest a close involvement and/or interaction of IL-35 with breast cancer cells, and also provides a basis for potential clinical intervention *via* manipulating/targeting IL-35 in the management of breast cancer.

Surprisingly, there is no significant correlation between IL-35 expression and TNM classification, nor with lymphatic metastasis in breast cancer patients (47). In addition, there is no significant difference between IL-35 expression within breast cancers and age (47). In the case of age, this may be due to the sample size that was investigated being relatively small, as well as the age cut-off used in the investigation being at 60 years. In terms of age, a 60-year cut-off isn’t the best for breast cancer female patients, who were almost all close to or post the menopause, and subsequently experienced less influence *via* sex hormone regulation. Similarly, the lack of correlation between IL-35 expression and tumour node and metastasis classification and metastasis was probably due to the relatively small sample size, with this study possibly being statistically underpowered. Thus, a study involving a larger sample size and multiple research centres should be performed to confirm the precise role of IL-35 during the development of breast cancer in the future.

However, by contrast, there is a controversial finding concerning IL-35 expression in colon cancer (50), which demonstrated that IL-35 is highly expressed within non-cancer mucosal tissue at the molecular and cellular levels, but that there was minimal IL-35 expression within the colon cancer tissue. Despite the low level of IL-35 expression within colon cancer compared to normal colonic mucosa, a significant correlation was still observed between relatively high IL-35 expression within colon cancer tissue and overall survival, or disease-free period (50), paradoxically suggesting a protective role of IL-35 during the development of colon cancer. This discrepancy in the role of IL-35 between breast and colon cancers may be related to the different functions of the organs, i.e. the breast epithelium usually exists in a germ-free environment; whereas the mucosal surface of the colon plays host to a large intestinal flora. These two completely different micro-environments within the breast and the colonic mucosa are likely to be a significant contributing factor to substantially different regulation, activation and/or recruitment of leucocytes, namely lymphocytes and macrophages, for the maintenance of local host immunity within these normal tissues. Subsequently, such different micro-environments will lead to different differential polarisation and actions of these leucocytes. The precise details of these processes require clarification in future studies.

The precise underlying mechanism of IL-35 activity in anti-tumour host immunity is not fully understood, but considering the role of naturally occurring Tregs (nTregs) and induced Tregs (iTregs) cells in response to IL-35 stimulation, it is reasonable to speculate that IL-35 suppresses host anti-tumour immunity *via* inhibiting cytotoxic T cells and/or antibody dependent mediated cytotoxicity in breast cancer (47). Thus, these data suggest that IL-35 contributes to the growth and/or differentiation of breast cancer during the development of the tumour in susceptible individuals. IL-35 regulates/suppresses host immunity *via* either natural Treg cells (51) or by further inducing conversion of T cells into induced Treg cells within the microenvironment. Thus, IL-35 plays both a paracrine as well as an autocrine function in regulating host immune status or maintaining homeostasis under normal conditions. From a pathogenesis point of view, previous data demonstrate that breast cancer cell–derived IL-35 is capable of enhancing tumour progression *via* induction of IL-35-producing induced regulatory T cells (47), and subsequently promotes tumour growth by enhancing myeloid cell accumulation and angiogenesis (52).

These data are also consistent with the finding that there is an inverse correlation between increased numbers of TAMs and reduced overall survival in breast cancer tissue, associated with a high level of angiogenesis (53), as well as metastasis (54), suggesting that TAMs promote the development of breast cancer. The possible mechanism by which TAMs act during the development of cancer has been elucidated in a murine breast cancer model, that showed that depletion of TAMs is the key regulator in inhibiting tumour growth *via* suppression of neovascularisation (55). This is further supported by a more recent study, showing that M2 TAMs augment triple negative breast cancer growth and promote angiogenesis *via* vascular endothelial growth factor (VEGF)/acutely transforming retrovirus (AKT)/mammalian target of rapamycin (mTOR) signalling pathway (56).

In conclusion, IL-35, an anti-inflammatory cytokine, augments the development of local breast cancer and boosts distant invasion and metastasis under an anti-inflammatory micro-environment *via* promoting M2 TAMs polarisation, as well as altering the function of Treg cells to regulate anti-cancer activities, in addition to the ability of IL-35 to directly and/or indirectly enhance angiogenesis. IL-35 may be a useful therapeutic target for precision medicine.

## IL-37 in breast cancer

Substantially reduced circulating IL-37 mRNA has been detected within breast cancer patients irrespective of metastasis, compared to that of healthy controls (57), suggesting that IL-37 is protective during the development of breast cancer. Interestingly, circulating IL-37 expression is highest for ER^+^/PR^+^/HER2^+^ breast cancer patients, compared to PR^+^ breast cancer, but not ER^+^/PR^+^ breast patients without metastasis (57), suggesting that IL-37 may also influence the prognosis *via* ER^+^/PR^+^/HER2^+^ signalling. However, there is no significant difference in circulating IL-37 mRNA expression among breast cancer patients with metastasis among the ER^+^/PR^+^/HER2^+^, ER^+^/PR^+^, and PR^+^ breast cancer patients (57), suggesting that IL-37 expression may be stage dependent, i.e. is more protective among the breast cancer patients without metastasis, but less effective on the patients with metastasis. The precise underlying mechanism of the IL-37 protective role during the development of breast cancer remains to be clarified.

Considering that IL-37 is an anti-inflammatory cytokine (34), IL-37 is able to suppress both innate and acquired immunity *via* inhibiting secretion of proinflammatory cytokines (58). It has been well documented that the pro- or anti-inflammatory responses either enhance or inhibit, respectively, the development of malignancy within the microenvironment (59). This is consistent with the observation that reduced circulating IL-37 mRNA expression is accompanied by reduced circulating CD8^+^ Tc cells within breast cancer patients (57). However, the number of TILs in breast cancer correlates with prognosis of overall survival (60), particularly following neoadjuvant chemotherapy. A possible explanation is that reduced circulating CD8^+^ Tc cells is due to increased recruitment of local CD8^+^ Tc cell infiltration in the breast tissue, suggesting that IL-37 may regulate host immunity *via* CD8^+^ Tc cells activity mediating direct and/or indirect cytotoxicity against breast cancer. This is consistent with the finding that there is a close correlation between the expression of IL-37 on dendritic cells and overall survival of hepatocellular carcinoma cells *in vivo* (43). However, the most critical issue is that there is no data available about the production of IL-37 in breast cancer tissue itself, which is rather urgently needed to confirm the role of IL-37 during the development of breast cancer *in vivo*.

From a functional point of view, a significant reduction has been observed in the development of breast cancer in immune competent BALB/c mice receiving IL-37β transgenic murine origin breast cancer cells, compared to that of non-transgenic breast cancer cells *in vivo* (61). Importantly, the suppression of the development of breast cancer is abrogated in recipients with T cell deficient BALB/c nude mice, in the presence of exogenous IL-37 administration, suggesting the important anti-tumour role of IL-37β is T cell mediated (61). This is consistent with the finding in human breast cancer, that has shown that the number of infiltrating CD8^+^ Tc cells in breast cancer correlates with prognosis of overall survival (60). In addition, considering that there is no mammary glands in BALB/c nude mice, NOD-SCID mice have been used as recipients, which lack all immune cells, including T and B lymphocytes, NK cells and macrophages (62), demonstrating that the protective role of IL-37 is ameliorated without host innate and acquired immunity in the NOD-SCID mice (61). This also correlates with the finding that IL-37 transfected peripheral blood mononuclear cells from hepatocellular carcinoma patients (23) promote M2 polarisation into M1 TAMs, subsequently inhibiting hepatocellular carcinoma cell proliferation *in vitro*, as well as in an animal model (nude mice) *via* the IL‐6/signal transducer and activator of transcription 3 (STAT3) signalling pathway. IL-37 has been shown to enhance anti-tumour activity in hepatocellular carcinoma *via* indirectly recruiting and activating dendritic cells *via* IL-12 and IL-18 (43), and subsequently *via* perforin and the FAS-L pathway (63).

Unfortunately, it remains to be explored which subset(s) of T cells, i.e. cytotoxic T cells, helper T cells or regulatory T cells, are involved in this anti-tumour activity in animals and/or human. In addition to T and B cells, other innate immune components, such as NK cells, macrophages and APCs may also be involved in the host immunity against the development of breast cancer.

We realise that breast and liver are both organs contain epithelial cells, which may share some similar biological/physiological properties, although there is a difference between breast cancer and hepatocellular carcinoma with different microenvironments and/or carcinogenesis. In addition, the data obtained in the hepatocellular carcinoma study were either from *in vitro* or xenograft *in vivo* studies, which may not reflect the conditions found in the real world. In addition, the breast cancer animal model is in the immune competent recipients, whereas hepatocellular carcinoma is in nude mice (lacking host immunity), which could substantially contribute to the different outcomes. Nevertheless, the observation from hepatocellular carcinoma invites speculation that the protective role of IL-37 in breast cancer is perhaps also to boost polarisation of M1 macrophages and subsequently inhibit the growth of breast cancer. The number and function of the subsets of M1 vs M2 macrophages in breast cancer will be determined in future experiments in humans and animals and will be explored as potential therapeutic target(s) for precision medicine.

Taken together, these data suggest IL-37 plays a critical protective role during the development of breast cancer *via* promoting M1 polarization and both the number and function of Tc cells in the breast cancer tissues. The finding may also offer a novel therapeutic target in the management of breast cancer.

In conclusion, IL-35 seems to promote the development of breast cancer; whereas IL-37 protects the host during the development of breast cancer. However, it remains to be explored what the precise underlying pathogenesis of these two important cytokines involves in the development of breast cancer. Our review provides some useful information for development of potential therapeutic targets in precision medicine.

## Author contributions

YM, HS, XYW, XN and YC wrote the manuscript. BH and SB revised the manuscript. XPW concept the manuscript. All authors contributed to the article and approved the submitted version.

## Funding

Basic Research Innovation Group Project of Gansu Province (No. 22JR5RA709): 2. Natural Science Foundation of Gansu Province (No. 20JR10RA381).

## Conflict of interest

The authors declare that the research was conducted in the absence of any commercial or financial relationships that could be construed as a potential conflict of interest.

## Publisher’s note

All claims expressed in this article are solely those of the authors and do not necessarily represent those of their affiliated organizations, or those of the publisher, the editors and the reviewers. Any product that may be evaluated in this article, or claim that may be made by its manufacturer, is not guaranteed or endorsed by the publisher.
